# Mycorrhizal response in crop versus wild plants

**DOI:** 10.1371/journal.pone.0221037

**Published:** 2019-08-08

**Authors:** Vasilis Kokkoris, Chantal Hamel, Miranda M. Hart

**Affiliations:** 1 Department of Biology, University of British Columbia, Okanagan campus, Kelowna, BC, Canada; 2 Quebec Research and Development Centre, Agriculture and Agri-Food Canada, Quebec, QC, Canada; Estacion Experimental del Zaidin, SPAIN

## Abstract

We proposed a theoretical framework predicting mutualistic outcomes for the arbuscular mycorrhizal (AM) symbiosis based on host provenance (crop versus wild). To test the framework, we grew two isolates of *Rhizoglomus irregulare* (commercial versus an isolate locally isolated), with five crop plants and five wild plants endemic to the region that co-occur with the locally sourced fungus. While inoculation with either isolate had no effect on plant biomass, it decreased leaf P content, particularly for wild plants. All plants associating with the commercial fungus had lower leaf P. Overall, our data shows that wild plants may be more sensitive to differences in mutualistic quality among fungal isolates.

## Introduction

Arbuscular mycorrhizal (AM) fungi are obligate root symbionts that provide a wide spectrum of benefits to their hosts, such as improved nutrient uptake and stress tolerance [1]. These benefits have led to their use as bio-fertilizers in agriculture and horticulture over the past 30+ years [2]. Consumer demand for AM fungal biofertilizers is growing; the number of companies producing inoculum have more than doubled in the past decade [3,4].

Despite early promise [5], inoculation by AM fungi does not always lead to improved plant performance. Even under controlled greenhouse conditions, failure to colonize is common [6,7] and in cases of successful colonization, effects range from negative [8–11], negligible [12,13] to significant yield increase [14]. Inoculation with AM fungi in the field is likewise inconsistent, ranging from yield increases [15–18] to no significant effect [19,20].

Most of our knowledge about host responses to inoculation by AM fungi is based on domesticated cultivars [17,18,21,22], meaning we have a poor understanding of how inoculants may affect local plant populations and communities if they disperse beyond the target plant community [23]. AM fungal inoculants may pose little threat to natural plant communities because most commercial inoculants comprise cosmopolitan species with worldwide distribution [24]. But there exists large intraspecific variation among conspecifics, [25–28], including mutualistic quality [29] and genetics [27,30,31]. Such variation may make it difficult to predict the functioning of these inoculants, even if conspecifics naturally co-occur.

Symbiotic outcomes for wild plants may differ from domesticated cultivars, leading to differential responses to inoculation. Because wild plants generally depend more on AM fungi compared to cultivars [32–35], symbiotic outcomes may be more pronounced (positive or negative) for wild plants compared to cultivars. This effect may be exacerbated by local adaptation between wild plants and soil biota as native plants respond more positively to local, versus exotic AM fungi [36,37]. Not only are local fungi adapted to local conditions [38,39], mutualisms are more beneficial when partners share evolutionary history [40]. Taken together, such differential responses to commercial fungal inoculum may result in less beneficial mutualisms for local plants if commercial inoculants become naturalized.

**Fig 1:**
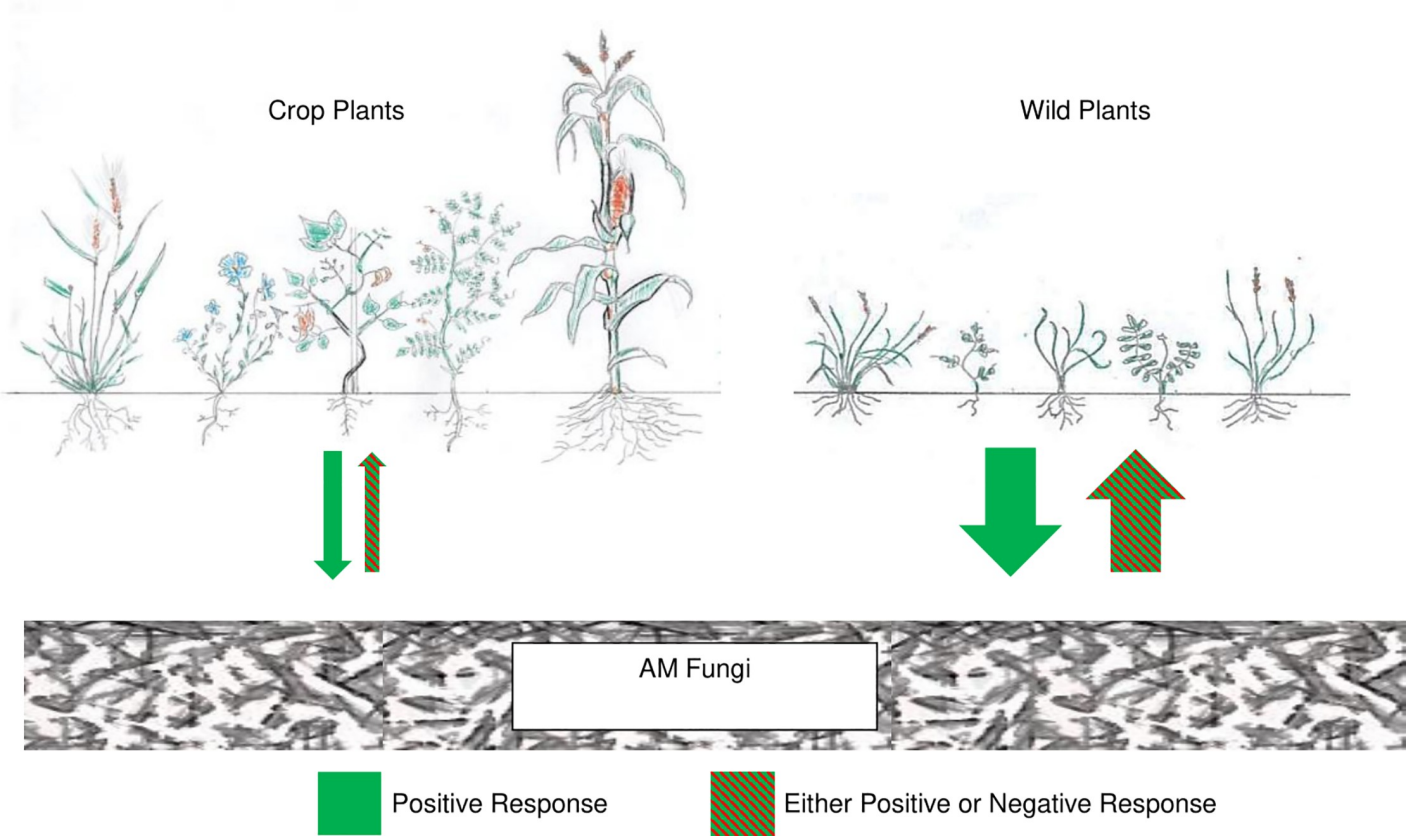
Experimental hypotheses and theoretical framework of differential response of wild and crop plants to AM fungi. The thickness of arrows corresponds to the magnitude of host-plant response. Inoculation effects on crop and wild plants (up arrows): We hypothesized inoculation with AM fungi would have a weak effect, either positive or negative, on crops due to lack of coadaptation [36,37] and reduced mycorrhizal responsiveness of domesticated plants [32–35]. For wild plants, we hypothesized effects of inoculation will be magnified, having a strong, either positive or negative effect, due to strong mycorrhizal responsiveness of wild plants [32–35]. Effects of plant provenance on AM fungi (down arrows): We hypothesized wild plants would have a strong positive effect on AM fungal growth due to increased dependency while crops would have a weak positive effect on AM fungi due to lack of coadaptation and reduced mycorrhizal responsiveness [36,37].

We designed a theoretical framework to predict how wild plants and crops can respond differently to AM fungal inoculation (Fig 1). To test the framework and to determine potential positive or negative impacts of commercial inoculants, we evaluated the mycorrhizal response of five wild plants and five crop plant species growing with a commercial AM fungal isolate and a locally sourced conspecific to test the questions:

- *Does plant provenance affect mycorrhizal response*?
- *Does plant provenance affect fungal response*?

## Materials & methods

### Experimental treatments

We tested the effect of host plant identity (five crop and five wild plant species) and AM fungal identity (commercial, locally sourced, non-mycorrhizal control) in a completely randomized block design (n = 8, total 240 experimental units). This experiment was conducted in greenhouse at UBC Okanagan from September 2015 to February 2016.

#### Plant identity

We tested the effect of host identity using “crop” and “wild” plants representing different functional groups (C_4_ grass, C_3_ grass, forb) that form AM symbioses (Table 1). These plants were selected to represent both common agricultural crops in the local area, and naturally occurring wild plants. Wild plant seeds were collected from wild populations near the source of the local inoculant. All wild plant seeds, as well as the flax (*Linum usitatissimum)*, lentils (*Lens culinaris)* and wheat (*Triticum aestivum)* seeds, were provided by Dr. M. Schellenberg from Semiarid Prairie Agricultural Research Centre, Agriculture and Agri-Food Canada in Swift Current, Saskatchewan. Corn (*Zea mays*) and soybean (*Glycine max)* seeds were obtained from West Coast Seeds Ltd.

**Table 1:** Cultivars and wild plants, from three distinct functional groups, that were inoculated with a commercial and a locally sourced isolate of *Rhizophagus irregularis*.

Functional group	Cultivars	Wild plants
C_4_ grass	*Zea mays* (Corn, var. Early Sunglow)	*Schizachyrium scoparium* (Little blue stem), *Calamovilfa longifolia* (Prairie sandreed)
C_3_ grass	*Triticum aestivum* (Lillian spring wheat)	*Agropyron dasystachyum* (Northern wheatgrass)
Forbs	*Linum usitatissimum* (Flax, var. Bethune), *Lens culinaris* (Lentils), *Glycine max* (Soybean, var. Kuroshinja Edamame)	*Hedysarum alpinum* (Alpine Sweetvetch), *Dalea candida* (White prairie clover)

#### Inoculant identity

*Rhizophagus irregularis* Schenck & Smith (DAOM 197198) (synonym *Glomus intraradices*, *G*. *irregulare* [41] and recently *Rhizoglomus irregulare* [42] was provided by BioSyneterra, Inc. (L'Assomption, Québec CANADA) This isolate has been cultivated in-vitro for more than 30 years (Stockinger, Walker & Schüßler, 2009) and is sold globally as a commercial inoculant. We also tested a locally sourced *Rhizophagus irregularis* (GD50, isolated in 2007 from SK (50° 34' 56.94" N/105° 29' 17.41" W), Agriculture and Agri-Food Canada, Swift Current Research and Development Centre). For the experiment, we used whole inoculum for both isolates (infected root fragments and spores), standardized based on propagule density per gram quantity. The propagule density for the locally sourced isolate inoculum was determined using the infection unit method by [43]and propagule density of the commercial isolate, as defined by the provider.

#### Growing conditions

Pots (3 L) were filled with 3.5 kg mix of sterile medium consisting of 75% medium-fine sand SAKRETE Play Sand and 25% Turface athletics MVP by volume. The medium was low in soil nutrients, as specified by the manufacturer's information, so we could control the nutrient status with fertilization. Turface consists of SiO2 (60%) Fe2O3 (5%) and other chemicals at less than 5% Al2O3, CaO, MgO, K2O, Na2O and TiO2 while sand had no nutrients. In each pot, we placed 26 propagules (8.7 g of inoculum containing infected root fragments and spores of the locally sourced isolate and 7 g similar inoculum of the commercial isolate). The inocula did not contain any additive nutrients and the carrier for both inocula was vermiculate. Three seeds were placed on top of the inoculum and covered with ~200 ml of growing medium, then thinned to one seedling per pot. Plants were watered with emitters supplying 2 l hr^-1^ to each pot, ~35 ml every day (1 minute per day) through the irrigation system and after 45 days the same amount of water as delivered every 2 days. Temperature ranged from 18 to 32°C.

A microbial wash from both the inoculants was applied at the beginning of the experiment to ensure that microbial community was same in all treatments and that any effect would be due to AM fungal isolate differences. This was made by adding 100 g of each inoculant to 4 l of water and mixing. The resulting solution was filtered through a 5μm mesh to exclude mycorrhizal fungal spores and infected roots.

Location of plants was randomized on greenhouse benches with each bench representing a ‘block’. The pots were subjected to 16 h light per day, with daily light integral (DLI) 71 μmol s^-1^ m^-2^ per μA measured with LI-250A light meter, Biosciences. Low P fertilizer (Miracle-gro 24-8-16) was added at half the manufacturer recommended dosage. Five ml of fertilizer was diluted in 8L of water. Fifty ml of that solution (containing 1.96 mg of P and 5.86 mg N) was applied every 14 days. Plants were grown for 16 weeks.

### Plant responses

#### Root, shoot, seed biomass and seed number

At harvest shoots were separated from roots. Fresh weight was measured before seeds and leaves were dried at 60°C for 48 h. A subsample of the root system was obtained for subsequent colonization measurements This subsample was included in the total root biomass value. After 48h at 60°C, dry weight of the roots was obtained. In addition to raw values, we calculated changes in biomass as Root: Shoot ratio. Only crops developed seeds during the experiment. Seeds were counted, then dried at 60°C for 48h for dry weight.

#### % Leaf P

Dried leaves were collected, pulverized and homogenized. % P in the leaves was calculated using a color development method, (using an acidified solution of ammonium molybdate, ascorbic acid and antimony) after acid digestion [44].

#### Mycorrhizal response (MR)

All plant responses were evaluated as mycorrhizal responsiveness. Mycorrhizal response (MR) represents the amount of benefit (if any) a plant gains from an AM fungal associate versus a nonmycorrhizal control [45]. For this study, we measured a) root: shoot ratio and b) % leaf P content. MR for root: shoot ratio was calculated for every plant species by the following formula:

$$MR=ln(\frac{i}{ii})$$

where ***ⅰ*** = root: shoot ratio of mycorrhizal plants and ***ⅱ*** = mean root: shoot ratio of non-mycorrhizal plants. MR for % leaf P content was calculated using the same formula but with % leaf P in lieu of root: shoot. To test for variability in response between crop and wild plants we used Leven’s test.

### Fungal responses

#### Root colonization

Roots were stained based on the protocol of [46]. Briefly, fresh roots were cut into 2-cm fragments and stained with Trypan blue. Fifteen to twenty root pieces were randomly collected and placed on a glass slide. The percentage of fungal organs (hyphae, vesicles and arbuscules) and the total root colonization were determined microscopically using the gridline intersect method of [47].

#### External mycelium and spores

For each pot, 100g of homogenized (wet) soil was used to determine external mycelia length as in [48]. A second 100 g (wet) soil sample was collected and dried. That sample was used to quantify spore density based on the protocol of (Gerdemann & Nicolson, 1963). Briefly, after recording the dry weight, each sample was placed into a blender and mixed in high speed for 5 seconds. The blended material was filtered through a series of sieves the final of which had an opening of 38 μm. After spores were transferred to 50 ml falcon tubes, centrifuged twice (at 1200 x g and 960 x g), and AM fungal spores were collected from the final supernatant in 50 ml falcon tubes. The number of spores was counted in each part of the grid.

### Statistical analysis

#### Does plant provenance affect mycorrhizal response?

We used a mixed effect linear model “lme4” version 1.1–12, Fitting Linear Mixed-Effects Models [49] to examine differences in mycorrhizal response for plants (crop vs. wild, fixed) exposed to different inoculation treatments (commercial inoculum vs. locally sourced inoculum vs. non-mycorrhizal control) with plant identity (random) and block (random). Data for all traits were logarithmically transformed to allow for normal distribution of residuals of the model. We examined biomass, % leaf P, MR of total biomass, root: shoot ratio and % leaf P. To test for equality of variances between crop and wild plants we used Levene’s test (“Rcmdr” version 2.4–4).

#### Does plant provenance affect fungal response?

Similar to above, we used a mixed effect linear model “lme4” to test for differences between plant treatments and AM fungal treatments on the fungal responses. Factors were, AM fungal isolate (fixed), plant provenance (crop vs. wild plants, fixed) with plant identity (random) and block (random). Data for all traits (except vesicle data) were logarithmically transformed to allow for normal distribution of residuals of the model. For vesicle data normalization of the residuals of the model was not possible, therefore we used a generalized mixed model (Poisson), which does not assume normality, with fixed and random factors as described above.

To proportionally represent the fungal traits (intra: extraradical) of each isolate the data were standardized using the “vegan” version 2.3–5 package (Community Ecology Package) [50]. Normalized trait values per isolate were summed and scaled to 100%. The ratio of intraradical to extraradical traits after normalization was calculated and we used a mixed effects linear model (lme4” version 1.1–12, Fitting Linear Mixed-Effects Models) [49]to examine the differences in trait investment strategies between the two isolates (commercial, locally sourced) among plant provenance (crop-wild, fixed), plant identity (10 species, random) and block (random).

R studio (Version 1.0.136–2009–2016 RStudio, Inc.) was used for all analyses.

## Results

### Does plant provenance affect mycorrhizal response?

#### Total biomass

**Fig 2:**
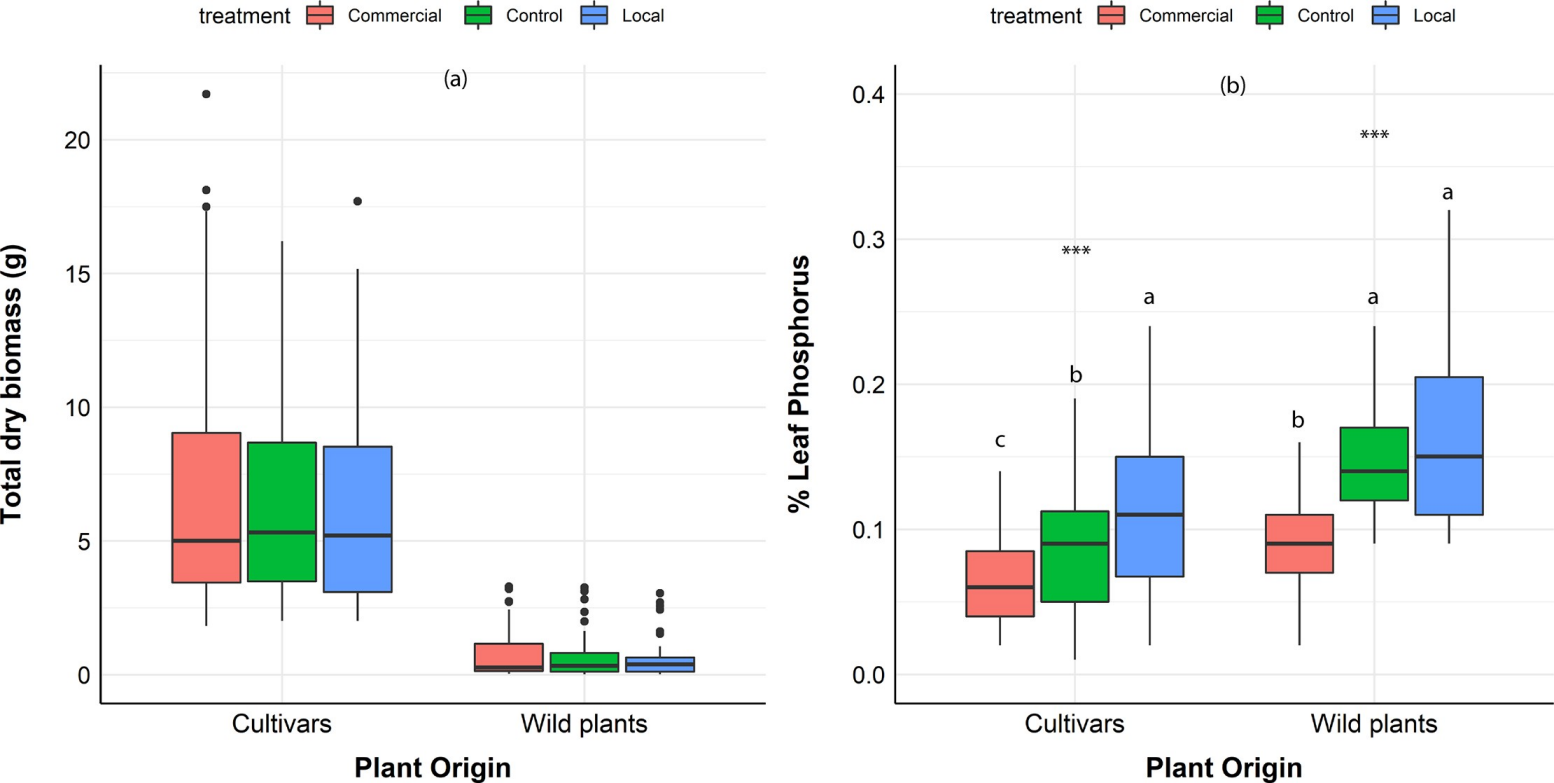
Fungal inoculation effects on cultivars and wild plants. Fungal inoculation effects on cultivars and wild plants: **(a)** Total plant biomass (g) **(b)** % leaf P. Red color boxplots: plants interacting with the commercial isolate, Blue color boxplots: plants interacting with the locally sourced isolate. Green color boxplots: plants without AM fungi. Box-plots show the third quartile and first quartile (box edges), median (middle line), range of the data (whiskers) and data outliers (black dots). *** p<0.001. Letters signify statistical significance (p<0.05) based on Tukey’s multiple comparisons test (agricolae version 1.2–3).

**Fig 3:**
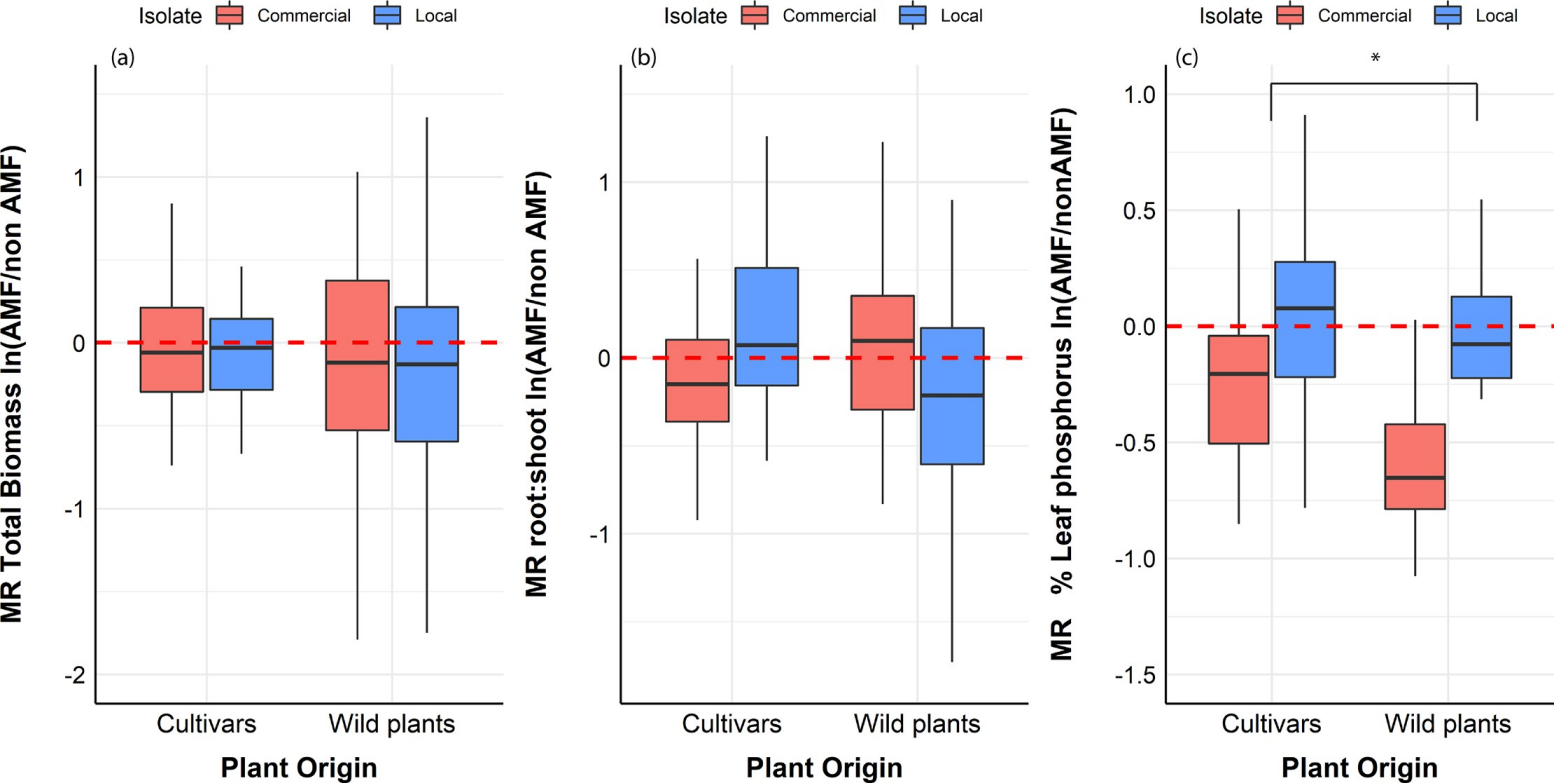
Mycorrhizal response in crop plants versus wild plants. **(a)** MR of total biomass of cultivars and wild plants when interacting with the commercial isolate (red) and the locally sourced isolate (blue). Red line indicates the mean value of nonmycorrhizal plants. **(b)** MR of root: shoot ratio of cultivars and wild plants when interacting with the commercial isolate (red) and the locally sourced isolate (blue). Red line indicates the mean value of the non-mycorrhizal plants. **(c)** MR of % leaf P of cultivars and wild plants when interacting with the commercial isolate (red) and the locally sourced isolate (blue). Red line indicates the mean value of the non-mycorrhizal plants. Box-plots show the third quartile and first quartile (box edges), median (middle line), range of the data (whiskers) and data outliers (black dots). * p<0.05. Mycorrhizal response (MR) represents the amount of benefit (if any) a plant gains from an AM fungal associate versus a nonmycorrhizal control.

**Fig 4:**
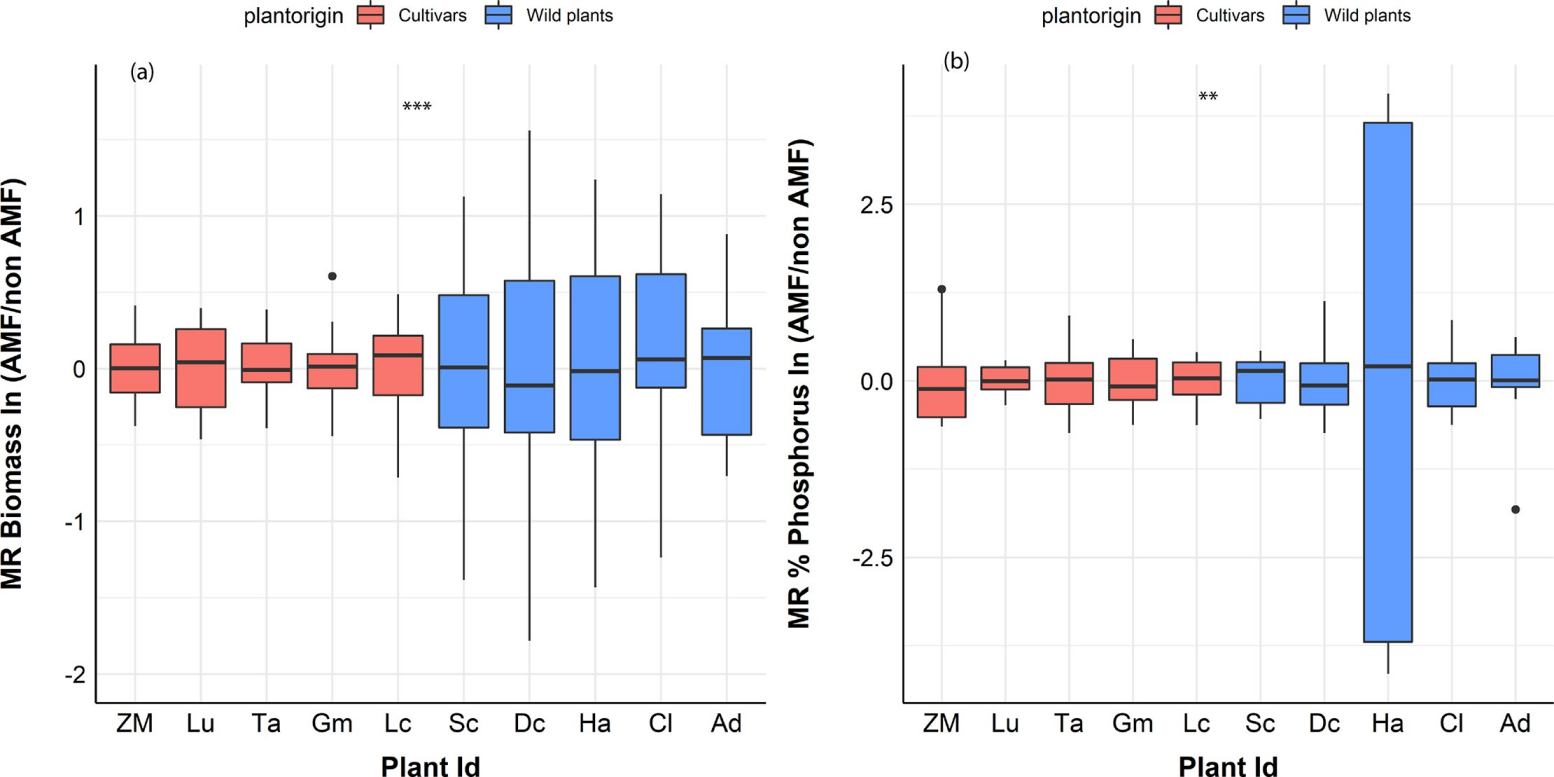
Mycorrhizal response variation in crop plants vs wild plants. **(a)** Biomass and **(b)** % leaf P. Red line indicates the mean value of the non-mycorrhizal plants. Crop are represented with red [ZM (Zea mays), LI (Linum usitatissimum), TA (Triticum aestivum), GM (Glycine max), LC (Lens culinaris)] and wild plants with blue color [(SC (Schizachyrium scoparium), DC (Dalea candida), HA (Hedysarum alpinum), CL (Calamovilfa longifolia), AD (Agropyron dasystachyum)]. Red line indicates the mean biomass of non-mycorrhizal plants. The third quartile and first quartile (box edges), median (middle line), and range of the data (whiskers) are shown. To test for equality of variance between crops and wild plant we used Levene’s test. Wild plants had significantly more variation compared to cultivars in terms of biomass p<0.001 and % P, p<0.01). Box-plots show the third quartile and first quartile (box edges), median (middle line), range of the data (whiskers) and data outliers (circles). ** p<0.01. *** p<0.001. Mycorrhizal response (MR) represents the amount of benefit a plant gains (if any) from an AM fungal associate versus a nonmycorrhizal control.

AM fungal identity did not affect total plant biomass compared to nonmycorrhizal controls for crop or wild plants (p = 0.51) (Fig 2A). Contrary to our hypothesis, wild plants were not more responsive to AM fungi in terms of biomass compared to crops overall (p = 0.89) (Fig 3A), but while examining individual responses, wild plants had significantly higher variation in their response to AM fungi in terms of biomass (Levene’s test, p<0.001) (Fig 4A). Individual plant biomass responses are presented in S1 Table.

#### Root: Shoot ratio

There was no difference among plants in root: shoot ratio (p = 0.63). Fungal identity had a significant effect on root: shoot ratio (p<0.01), that is the commercial AM isolate leading to larger root: shoot ratio compared to locally sourced AM isolate. There was a significant interaction between fungal identity and plant provenance (p<0.001) with locally sourced inoculum leading to increased root: shoot ratio for crops and decreased root: shoot ratio for wild plants (Fig 3B). Individual plant root: shoot ratio responses are presented in S1 Table.

#### % Leaf P

There was a significant interaction between fungal identity and plant provenance, with wild plants having a significant less % P than crop for the commercial AM fungus (p<0.05) (Fig 3C). AM fungal identity significantly affected plant % leaf P (p<0.001).

The commercial isolate reduced plant % leaf P levels (p<0.001) across all plants and plant provenance compared to control and locally sourced inoculum (Fig 2B). The locally sourced isolate increased crop % leaf P compared to control (p<0.05) but did not affect % leaf P of wild plants compare to control (p = 0.71) (Fig 2B). Wild plants had significantly higher variation in their response to AM fungi in terms of % leaf P (Levene’s test p<0.01) (Fig 4B). Individual plant % leaf P responses are presented in S1 Table.

### Does plant provenance affect fungal response?

#### Root colonization

**Fig 5:**
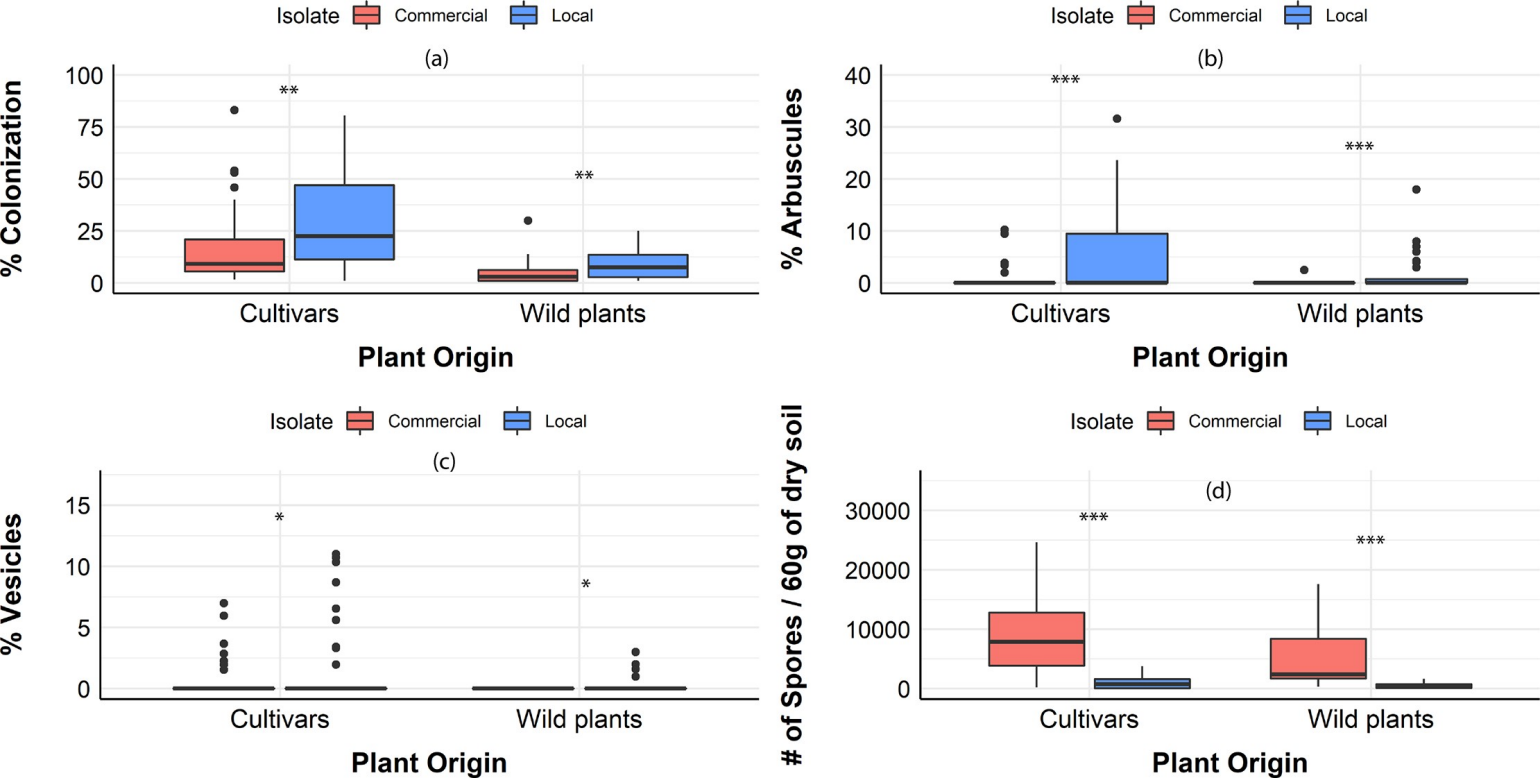
**Fungal response when associating cultivars or wild plants with a commercial isolate (red) or a locally sourced isolate (blue).** (a) per cent colonization, (b) per cent arbuscules, (c) per cent vesicles, (d) spore number per 60 gr of substrate (dry). Box-plots show the third quartile and first quartile (box edges), median (middle line), range of the data (whiskers) and data outliers (circles). * p<0.05, ** p<0.01 *** p<0.001.

Wild plants had lower colonization for both isolates compared to the crops (p<0.01) (Fig 5A). No AM colonization was observed in non-mycorrhizal controls. The commercial isolate had lower root colonization compared to the locally sourced isolate (p<0.001) (Fig 5A). For individual host % colonization data please see S2 Table.

#### Arbuscules

In general, wild plants had fewer arbuscules compared to crops (p<0.01) (Fig 5B). The commercial isolate formed fewer arbuscules compared to the locally sourced isolate (p<0.001) The number of arbuscules differed significantly among the two AM fungal isolates (Fig 5B).

#### Vesicles

Fewer vesicles were observed for the wild plants compared to crops regardless of fungal isolate (p<0.05) (Fig 5C). The number of vesicles differed significantly among the two AM fungal isolates in all hosts (p<0.05) (Fig 5C). The commercial isolate formed fewer vesicles compared to the locally sourced isolate

#### Spores

Sporulation was not influenced by the plant provenance (p = 0.33) (Fig 5E). However, spore density differed significantly between fungal isolates. The commercial isolate produced more spores compared to the locally sourced isolate (p<0.001) (Fig 5D).

#### Extraradical mycelium (ERM) length

There was no difference in extraradical mycelium length between wild plants and crops (p = 0.34) nor between the fungal isolates (p = 0.58).

#### Allometry: Proportional fungal trait distribution (Intraradical traits/extraradical traits)

**Fig 6:**
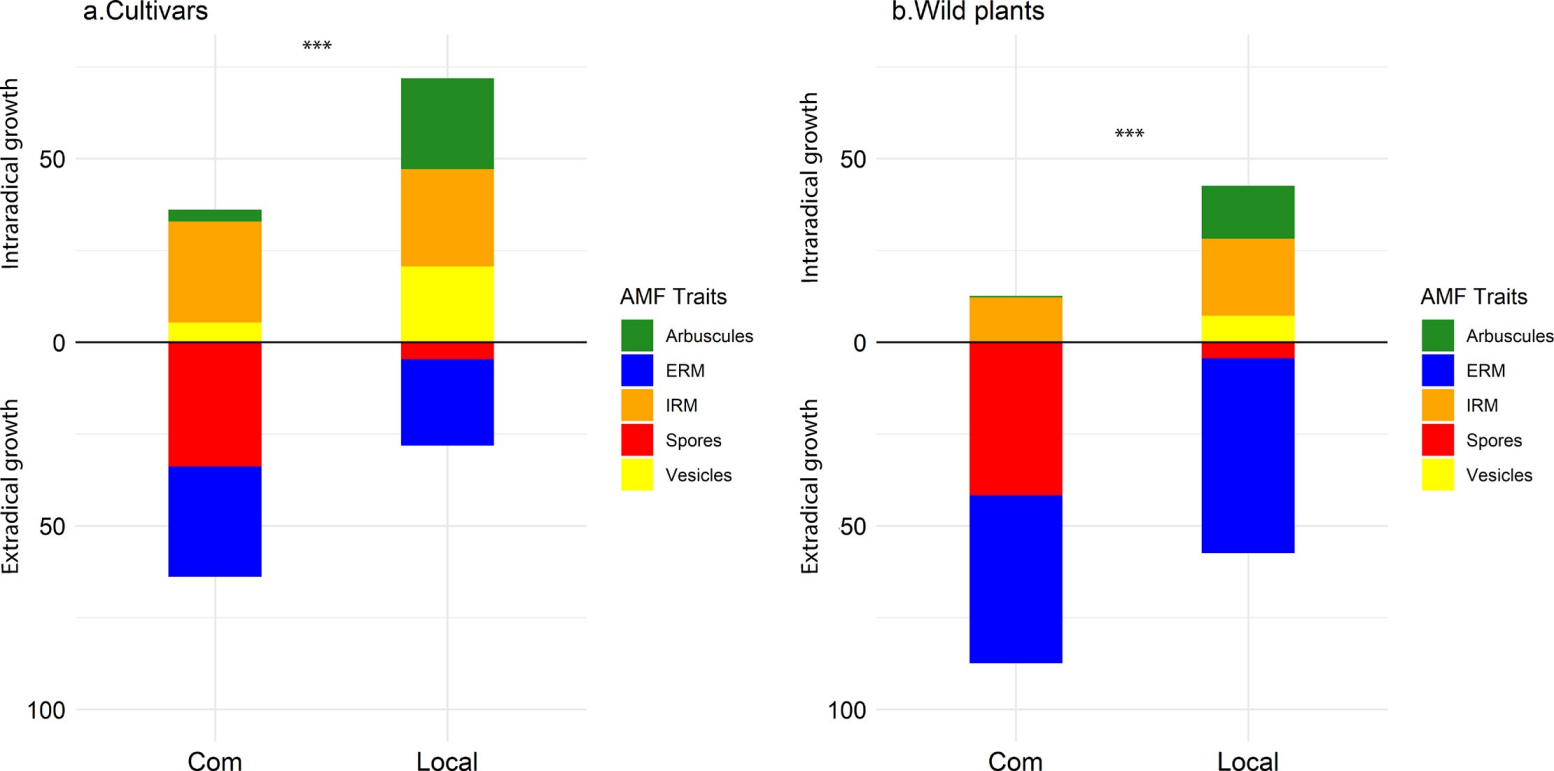
Proportional representation of fungal structures in crop and wild plants. Arbuscules (green), extraradical mycelium (blue), intraradical mycelium (orange), spores (red) and vesicles (yellow) of the commercial (Com) and local isolate (Local) when associated with **(a)** cultivars and **(b)** wild plants. *** p<0.001.

Wild plants had a lower intraradical: extraradical ratio compared to crops (p<0.01) (Fig 6). There was a significant difference at the ratio of intraradical to extraradical traits between the two AM fungi (p<0.001). The commercial isolate invested more extraradically compared to the locally sourced isolate.

## Discussion

Our experiment provides preliminary evidence that introduced fungi may negatively affect AM mutualistic outcomes. Wild plants had pronounced variation in their response to fungi compared to crops. Responses ranged from positive to strongly negative revealing the sensitivity of wild plants to fungal identity, even to isolates within the same fungal species. Future studies assessing the risk of fungal inoculants should examine this relationship on a variety of hosts, multiple functional groups and fungal isolates.

### Plant responses

While wild plants have been reported to be more responsive to AM fungi [32–35], we did not find support for this in terms of **biomass** when looking at plants as either ‘crop’ or ‘wild’ plants. Because our wild plants were perennials, it is possible that our study did not allow enough time for full biomass differences to manifest, as the study ended when crops, but not wild plants, had senesced. Thus, our inability to detect a difference among crops may have been due to time constraints [13].

When we looked at responses of individual plant species, wild plants varied more in their response to inoculation with AM fungi, ranging from highly negative to highly positive. Variation in biomass has been documented for wild plants in the literature, particularly, for perennials versus annuals [51], and natives versus exotics [40] revealing the strong mycorrhizal responsiveness of wild plants.

Inoculation with the commercial isolate led to increased **shoot: root** for crops but not for wild plants. It is not uncommon to observe alteration in root: shoot ratio with inoculation by AM fungi [52–54]. The “functional equilibrium” theory suggests that plants allocate biomass preferentially to maximize resource acquisition, a plant should favor above ground growth when carbon is limited. Because carbon allocation from plant to fungus can lead to carbon limitation [55], our results indicate that the commercial isolate may have posed more of a carbon demand than the local isolate, leading to increased shoot allocation. Such changes may lead to reduced nutrient acquisition for plants associated with the commercial fungus in some conditions.

We found support for our hypothesis that plant provenance affects mycorrhizal response in terms of **% leaf P**. Wild plants, surprisingly, experienced a decrease in percent % leaf P when inoculated with AM fungi. While there is evidence for wild plants as more mycorrhizal dependent compared to crops [32–35], their increased sensitivity to AM fungi can lead to magnified negative effects as well particularly when fungi and plants are competing for limited resources [40].

In our study, the commercial isolate was less mutualistic in terms of % leaf P and this effect was magnified in wild plants. Other studies have shown of AM fungal inoculation leading to reduced host P [8–11]. While such reductions may be related to greenhouse growing conditions, reduced P following inoculation may also indicate a less mutualistic AM association in some cases [10,56,57]. In our study, plants inoculated with the commercial isolate had lower P compared to non-mycorrhizal controls which could indicate either direct competition between plant and fungus for P, or P hoarding by the fungus [58]. It may also mean that the commercial isolate does not have enhanced P uptake ability over plant-direct uptake routes, perhaps through loss of traits during domestication. Further studies comparing more isolates with isotope labelling and genomic studies could elucidate the mechanism involved.

Nevertheless, in order to further examine wild and cultivar plant responses to inoculation, that will more accurately represent natural growing conditions, it is very important for future studies to consider soil physicochemical properties, since it has been shown that sympatric combinations of plants, fungi and soil, can lead to increased MR [37].

### Fungal responses

We did not find support for our hypothesis that wild plants were more beneficial to AM fungi compared to crops. Rather, large differences in growth strategies among the two fungal isolates may explain differences plant response. The commercial isolate in our study had few **arbuscules** at time of harvest. This is unusual as arbuscules (or coils) are considered fundamental to the mutualism under natural conditions [59]. Reduction of arbuscules has been reported for a variety of AM fungal species (including *Rhizophagus sp*.) under stressful environments [60,61], and due to differences in harvest time and level of fertilization [62,63]. Specifically, suppression of arbuscules can occur with increasing P or N [62] and changes in arbuscule formation due to time of harvest can be regulated by the species identity [63]. In our experiment, differences are likely do to fungal strategies since there was no suppression of arbuscules in the locally sourced isolate.

Low levels of arbuscules in the commercial isolate may be explained by considering the conditions under which the commercial isolate is propagated. Large-scale inoculum production occurs mostly on transformed roots, which are able to directly uptake most of their resources from the nutrient medium [64] and have very low nutrient requirements [65]. Such a luxurious in nutrients environment, may require fewer arbuscules or enhance the resource sink abilities of the isolate, but this remains to be seen. Given that there is still considerable debate over the function of arbuscules [66,67], it is difficult to identify factors that promote or suppress their production.

Alternatively, propagation using transformed roots for inoculum production may favor ruderal behavior since ruderal traits, such as rapid growth and early production of abundant propagules, are of interest to inoculum production industry [68]. If the commercial isolate is more ruderal, the extremely low number of arbuscules observed for the commercial isolate at harvest might be due to a faster`completion of its life cycle compared to the locally sourced isolate. Future studies examining the progress of the symbiosis over time would be able to reveal such significant differences in life history strategies.

Overall, we observed low values of **root colonization** for both isolates across all plants. Our experiment was conducted during the winter. Thus, it is possible our plants reduced the amount of carbon allocated to the roots and subsequently to AM fungi [69].

While there was no difference in the extent of **ERM** among fungal isolates, the commercial isolate invested heavily in **spore production** compared to the local isolate. Large differences in spore production among isolates is not unusual, as there have been many reports of inter and intraspecific variation in fungal traits, over several orders of magnitude in some cases [28,54,70–72]. Nevertheless, the difference in sporulation rate observed in this study, is unusually large (50x) and represents a significant carbon drain for hosts associating with this fungus.

Low levels of root colonization during winter growing conditions and especially due to light limitations is typical ([73–76]) but in our study it had the unintended benefit of potentially exacerbating differences in LHS among our isolates. Under the carbon limiting conditions of our study, the commercial isolate allocated resources into non-mutualistic structures (i.e. spores)–thereby competing with its host for nutrients. In contrast, the wild isolate allocated resources to mutualistic structures (i.e. arbuscules). It would be interesting to test whether such strategies are a response to resource levels, or robust across gradients.

### Allometry (Intraradical: Extraradical investment)

Root colonization is not a good predictor of symbiosis quality [77,78]. On the contrary, examining specific traits can be more meaningful [79,80]. The commercial isolate had a significantly different growth pattern compared to the locally sourced isolate that was consistent among hosts and plant provenance, revealing important LHS variations between the two isolates. The commercial isolate had a high soil biomass, which could enhance soil exploration potential and subsequently, host benefit [81]. Considering the differences in spore number between the two isolates, deriving from the same quantity of ERM, means that the commercial isolate represented a nutrient sink rather than a source (including C and P).

## Conclusions

Wild plants had highly variable response to inoculation by AM fungi compared to crop plants. This raises concerns about how inoculation practices may affect wild plant/soil communities. Our study provides evidence that the commercial isolate used in this study may be less mutualistic under some conditions. The commercial isolate invested in spore production at the expense of intraradical structures, suggesting a more “selfish” strategy. Correspondingly, plants experienced decreased P with the commercial isolate. It is important for future studies to consider fitness consequences associated with inoculation studies, as poor mutualists may not be apparent over one generation. Considering the number of propagules produced by the commercial isolate, there is a high likelihood of spread beyond the agricultural fields displacing native AM fungi. Future studies should focus on the viability and establishment of these propagules beyond agricultural systems.

## Supporting information

S1 Table Do AM fungal isolates affect plants differentially? Descriptive statistics along with Analysis of variance (ANOVA) and Tukey’s multiple comparisons test (agricolae version 1.2–3). Effects of inoculation treatments (Control, commercial AMF, locally sourced AMF) on the five crop and five wild plants. The values are reported as: mean ± SD {MR}. MR (Mycorrhizal response) as described by Baon et al. [45] (100*(M- NM)/NM) by using mean values for M and NM. Positive MR is colored green while negative MR is colored red.
(DOCX)

S2 Table Descriptive statistics of percent root colonization. Percent root colonization of the two isolates (commercial AMF, locally sourced AMF) on the five crop and five wild plants.
(DOCX)

S3 Table Plant and fungal growth raw data are available as supplemental materials. (XLSX)
