# Supplementary material for: Mycorrhizal response in crop versus wild plants
Source: PLoS One. 2019 Aug 8;14(8):e0221037. doi: 10.1371/journal.pone.0221037 (PMC6687160; doi:10.1371/journal.pone.0221037)
Supplement: S1 Table — Descriptive statistics along with Analysis of variance (ANOVA) and Tukey’s multiple comparisons test (agricolae version 1.2–3). Effects of inoculation treatments (Control, commercial AMF, locally sourced AMF) on the five crop and five wild plants. The values are reported as: mean ± SD {MR}. MR (Mycorrhizal response) as described by Baon et al. [45] (100*(M- NM)/NM) by using mean values for M and NM. Positive MR is colored green while negative MR is colored red. (DOCX) [file pone.0221037.s001.docx]

| Plant identity | Treatment | Shoot Biomass dry (g) | Root Biomass dry (g) | Total Biomass dry (g) | Root: shoot Ratio | Leaf P % |
| --- | --- | --- | --- | --- | --- | --- |
| *Zea mays* (Corn) | **Control** | 13.06±2.82^a^ {NA} | 1.27±0.70^a^ {NA} | 14.33±3.05^b^ {NA} | 0.10±0.05^a^ {NA} | 0.04±0.02^a^ {NA} |
|  | **Com** | 15.48±3.91^a^ {18.5%} | 1.62±0.56^a^ {27.6%} | 17.10±4.40^a^ {19.3%} | 0.10±0.01^a^ {0%} | 0.03±0.01^a^ {-25%} |
|  | **Local** | 13.47±2.78^a^ {3.14%} | 2.12±1.32^a^ {66.9%} | 15.59±2.94^ab^ {8.8%} | 0.17±0.14^a^ {70%} | 0.06±0.05^a^ {50%} |
| ANOVA |  | F=3.30, **p=0.07** | F=2.47, p= 0.12 | F=4.57, **p=0.03*** | F=1.50, p=0.25 | F=1.30, p=0.30 |
| *Linum usitatissimum* (Flax) | **Control** | 3.26±0.78^a^ {NA} | 0.43±0.29^a^ {NA} | 3.69±0.97^a^ {NA} | 0.13±0.06^b^ {NA} | 0.18±0.04^ab^ {NA} |
|  | **Com** | 2.85±0.97^a^ {-12.6%} | 0.31±0.16^a^ {-27.9%} | 3.16±1.11^a^ {-17.5%} | 0.10±0.04^ab^ {-23.1%} | 0.15±0.02^b^ {-16.7%} |
|  | **Local** | 2.60±0.74^a^ {-20.3%} | 0.71±0.70^a^ {65.1%} | 3.31±1.25^a^ {-10.3%} | 0.25±0.16^a^ {92.3%} | 0.20±0.03^a^ {11.1%} |
| ANOVA |  | F=3.19, **p=0.07** | F=3.46, **p=0.06** | F=1.04, p=0.38 | F=6.24, **p=0.01*** | F=6.19, **p=0.01*** |
| Triticum aestivum (Lillian wheat) | **Control** | 8.22±2.26^a^ {NA} | 0.79±0.51^a^ {NA} | 9.02±2.59^a^ {NA} | 0.09±0.05^a^ {NA} | 0.07±0.03^a^ {NA} |
|  | **Com** | 7.87±1.63^a^ {-4.3%} | 0.52±0.22^a^ {-34.2%} | 8.39±1.82^a^ {-7%} | 0.06±0.02^a^ {-33.3%} | 0.05±0.03^a^ {-28.6%} |
|  | **Local** | 7.32±1.37^a^ {-11%} | 0.72±0.38^a^ {-8.86%} | 8.04±1.63^a^ {-10.9%} | 0.09±0.04^a^ {0%} | 0.07±0.03^a^ {0%} |
| ANOVA |  | F=1.09, p=0.36 | F=0.9, p=0.42 | F0.87, p=0.43 | F=1.32, p=0.29 | F=1.53, p=0.25 |
| *Glycine max* (Soya beans) | **Control** | 3.57±1.49^a^ {NA} | 0.22±0.15^a^ {NA} | 3.49±1.60^a^ {NA} | 0.06±0.02^a^ {NA} | 0.09±0.02^ab^ {NA} |
|  | **Com** | 4.90±2.30^a^ {37.3%} | 0.27±0.15^a^ {22.7%} | 5.17±2.42^a^ {48.1%} | 0.06±0.02^a^ {0%} | 0.07±0.02^b^ {-22.2%} |
|  | **Local** | 4.00±1.00^a^ {12.1%} | 0.32±0.10^a^ {45.5%} | 4.32±1.03^a^ {23.8%} | 0.08±0.03^a^ {33.3%} | 0.11±0.03^a^ {22.2%} |
| ANOVA |  | F=2.27, p=0.14 | F=1.42, p=0.28 | F=2.20, p=0.15 | F=2.04, p=0.17 | F=8.19, **p=0.004**** |
| *Lens culinaris* (Lentils) | **Control** | 4.78±1.34^a^ {NA} | 0.64±0.45^a^ {NA} | 5.42±1.71^a^ {NA} | 0.12±0.07^a^ {NA} | 0.09±0.03^ab^ {NA} |
|  | **Com** | 4.32±1.26^a^ {-9.6%} | 0.40±0.16^a^ {-37.5%} | 4.73±1.35^a^ {-11.8%} | 0.09±0.04^a^ {-25%} | 0.06±0.01^b^ {-33.3%} |
|  | **Local** | 4.43±1.81^a^ {-7.3%} | 0.59±0.34^a^ {-7.8%} | 5.01±2.06^a^ {-7.6%} | 0.13±0.06^a^ {8.3%} | 0.11±0.02^a^ {22.2%} |
| ANOVA |  | F=0.28, p=0.76 | F=1.55 p=0.25 | F=0.47, p=0.63 | F=1.14, p=0.35 | F=9.48, p**=0.003**** |
| *Schizachyrium scoparium* (Little blue stem) | **Control** | 0.61±0.34^a^ {NA} | 0.18±0.11^a^ {NA} | 0.80±0.45^a^ {NA} | 0.30±0.12^a^ {NA} | 0.12±0.02^a^ {NA} |
|  | **Com** | 0.77±0.52^a^ {26.2%} | 0.18±0.12^a^ {0%} | 0.95±0.54^a^ {18.8%} | 0.38±0.56^a^ {26.7%} | 0.06±0.02^b^ {-50%} |
|  | **Local** | 0.37±0.28^a^ {-39.3%} | 0.15±0.07^a^ {-16.7%} | 0.51±0.27^a^ {-36.3%} | 0.70±0.61^a^ {133.4%} | 0.10±0.08^a^ {-16.7%} |
| ANOVA |  | F=2.06, p=0.16 | F=0.37, p=0.70 | F=2.05, p=0.16 | F=1.84, p=0.19 | F=27.98, p**<0.001***** |
| *Dalea candida* (White prairie clover) | **Control** | 0.16±0.13^a^ {NA} | 0.07±0.04^a^ {NA} | 0.22±0.17^a^ {NA} | 0.51±0.39^a^ {NA} | 0.30±0.20^a^ {NA} |
|  | **Com** | 0.13±0.10^a^ {-18.8%} | 0.09±0.07^a^ {22.2%} | 0.22±0.16^a^ {0%} | 0.65±0.40^a^ {27.5%} | 0.14±0.02^a^ {-53.3%} |
|  | **Local** | 0.20±0.20^a^ {25%} | 0.12±0.11^a^ {71.4%} | 0.32±0.32^a^ {45.5%} | 0.60±0.26^a^ {17.6%} | 0.33±0.17^a^ {10%} |
| ANOVA |  | F=0.73, p=0.49 | F=0.79, p=0.47 | F=0.70, p=0.51 | F=0.63, p=0.70 | F=3.05, **p=0.07** |
| *Hedysarum alpinum* (Alpine Sweetvetch) | **Control** | 0.27±0.32^a^ {NA} | 0.15±0.16^a^ {NA} | 0.42±0.48^a^ {NA} | 0.83±0.45^a^ {NA} | 0.18±0.05^a^ {NA} |
|  | **Com** | 0.31±0.31^a^ {14.8%} | 0.29±0.19^a^ {93.3%} | 0.60±0.60^a^ {42.9%} | 1.70±1.20^a^ {104.8%} | 0.10±0.05^b^ {-44.4%} |
|  | **Local** | 0.33±0.19^a^ {22.2%} | 0.28±0.08^a^ {86.7%} | 0.61±0.19^a^ {48.8%} | 1.51±1.65^a^ {81.9%} | 0.17±0.03^a^ {-5.6%} |
| ANOVA |  | F=0.75, p=0.49 | F=1.48, p=0.26 | F=1.62, p=0.23 | F=1.62, p=0.23 | F=5.62, **p=0.01*** |
| *Calamovilfa longifolia* (Prairie sandreed) | **Control** | 0.25±0.14^a^ {NA} | 0.15±0.09^a^ {NA} | 0.41±0.21^a^ {NA} | 0.75±0.41^a^ {NA} | 0.15±0.02^a^ {NA} |
|  | **Com** | 0.30±0.18^a^ {20%} | 0.17±0.17^a^ {13.3%} | 0.46±0.34^a^ {12.2%} | 0.51±0.26^a^ {-32%} | 0.09±0.02^b^ {-40%} |
|  | **Local** | 0.27±0.21^a^ {8%} | 0.15±0.15^a^ {0%} | 0.41±0.33^a^ {0%} | 0.55±0.42^a^ {-26.6%} | 0.18±0.06^b^ {20%} |
| ANOVA |  | F=0.09, p=0.91 | F=0.04, p=0.95 | F=0.30, p=0.74 | F=0.96, p=0.40 | F=14.65, **p<0.001***** |
| *Agropyron dasystachyum* (Northern wheatgrass) | **Control** | 2.14±0.90^a^ {NA} | 1.33±0.88^a^ {NA} | 3.47±1.70^a^ {NA} | 0.57±0.24^a^ {NA} | 0.14±0.02^a^ {NA} |
|  | **Com** | 2.41±0.75^a^ {12.6%} | 1.15±0.56^a^ {-13.5%} | 3.56±1.27^a^ {2.6%} | 0.45±0.11^a^ {-21.1%} | 0.09±0.03^a^ {-35.7%} |
|  | **Local** | 1.99±0.80^a^ {-7%} | 0.90±0.54^a^ {-32.3%} | 2.88±1.28^a^ {-17%} | 0.45±0.18^a^ {-21.1%} | 0.15±0.03^a^ {7.1%} |
| ANOVA |  | F=0.72, p=0.50 | F=0.94, p=0.41 | F=0.68, p=0.52 | F= 1.08, p=0.36 | F=5.36, **p=0.01*** |
