# Supplementary material for: Mycorrhizal response in crop versus wild plants
Source: PLoS One. 2019 Aug 8;14(8):e0221037. doi: 10.1371/journal.pone.0221037 (PMC6687160; doi:10.1371/journal.pone.0221037)
Supplement: S2 Table — Percent root colonization of the two isolates (commercial AMF, locally sourced AMF) on the five crop and five wild plants. (DOCX) [file pone.0221037.s002.docx]

| Plant ID | Fungal isolate | min | Q1 | median | Q3 | max | mean | SD | n |
| --- | --- | --- | --- | --- | --- | --- | --- | --- | --- |
| *Zea mays*  (Corn) | Commercial | 6.67 | 10.03 | 17.22 | 29.30 | 53.00 | **21.45** | 15.65 | 8.00 |
|  | Locally sourced | 10.62 | 12.47 | 17.90 | 49.47 | 77.27 | **31.56** | 25.62 | 8.00 |
| *Linum usitatissimum*  (Flax) | Commercial | 3.00 | 6.72 | 14.64 | 30.89 | 54.00 | **21.37** | 19.50 | 8.00 |
|  | Locally sourced | 1.00 | 20.66 | 29.45 | 38.60 | 80.56 | **32.59** | 24.43 | 8.00 |
| *Triticum aestivum*  (Lillian wheat) | Commercial | 3.00 | 7.00 | 14.00 | 21.25 | 40.00 | **16.50** | 12.39 | 8.00 |
|  | Locally sourced | 2.00 | 3.72 | 11.14 | 49.79 | 58.97 | **24.16** | 25.18 | 8.00 |
| *Glycine max*  (Soya beans) | Commercial | 1.50 | 3.56 | 5.84 | 10.77 | 83.08 | **15.60** | 27.49 | 8.00 |
|  | Locally sourced | 2.00 | 17.17 | 28.59 | 34.63 | 53.00 | **27.87** | 17.37 | 8.00 |
| *Lens culinaris*  (Lentils) | Commercial | 1.56 | 3.44 | 4.95 | 7.52 | 14.77 | **6.40** | 4.64 | 8.00 |
|  | Locally sourced | 9.09 | 14.99 | 19.31 | 26.81 | 56.09 | **23.43** | 14.96 | 8.00 |
| *Schizachyrium scoparium* (Little blue stem) | Commercial | 1.00 | 2.00 | 2.50 | 3.00 | 7.00 | **2.87** | 1.81 | 8.00 |
|  | Locally sourced | 8.00 | 11.25 | 16.50 | 19.00 | 22.00 | **15.25** | 5.12 | 8.00 |
| *Dalea candida*  (White prairie clover) | Commercial | 0.99 | 1.00 | 1.01 | 2.65 | 6.25 | **2.10** | 1.86 | 8.00 |
|  | Locally sourced | 1.00 | 2.00 | 6.00 | 13.00 | 25.00 | **8.75** | 8.55 | 8.00 |
| *Hedysarum alpinum* (Alpine Sweetvetch) | Commercial | 1.00 | 1.05 | 3.01 | 5.29 | 6.74 | **3.37** | 2.35 | 8.00 |
|  | Locally sourced | 1.00 | 1.00 | 1.50 | 2.50 | 11.00 | **2.88** | 3.44 | 8.00 |
| *Calamovilfa longifolia* (Prairie sandreed) | Commercial | 2.00 | 4.75 | 7.28 | 13.18 | 30.00 | **10.29** | 8.96 | 8.00 |
|  | Locally sourced | 3.00 | 10.00 | 12.50 | 15.25 | 22.87 | **12.48** | 5.97 | 8.00 |
| *Agropyron dasystachyum* (Northern wheatgrass) | Commercial | 1.00 | 1.75 | 2.97 | 4.75 | 12.00 | **4.12** | 3.72 | 8.00 |
|  | Locally sourced | 1.00 | 3.00 | 4.50 | 7.00 | 8.00 | **4.75** | 2.55 | 8.00 |
